# Dryocrassin ABBA Inhibits the Function of LLO and SortaseA to Alleviate the Virulence of *Listeria monocytogenes*

**DOI:** 10.4014/jmb.2510.10021

**Published:** 2026-01-26

**Authors:** Jiahui Lu, Junlu Liu, Hanbing Zhou, Yifan Duan, Zehua Wang, Guizhen Wang

**Affiliations:** College of Biological and Food Engineering, Jilin Engineering Normal University, Changchun 130052, P.R. China

**Keywords:** Anti-virulence, Anti-infection, Sortase A, LLO, Dryocrassin ABBA

## Abstract

*Listeria monocytogenes* is a deadly foodborne pathogen, its virulence factors Listeriolysin O (LLO) and Sortase A (SrtA) play a critical role in its infection and have been identified as key targets for developing its inhibitor. This study reveals that the natural compound Dryocrassin ABBA (ABBA) alleviates the toxicity of *L. monocytogenes* by effectively inhibiting the functions of LLO and SrtA. ABBA bound to the binding pocket of LLO and inhibited the formation of its oligomers, which in turn reduced the ability of LLO to lyse mammalian erythrocytes. When co-cultured with *L. monocytogenes*, ABBA reduced the hemolytic activity of the cultural supernatant. In addition, ABBA bound to SrtA and reduced its transpeptide activity, which in turn resulted in a reduction in the formation of bacterial biofilm, the adhesion and invasion of *L. monocytogenes* to cells. Thr415 and Lys505 of LLO, and Asn92 and Pro220 of SrtA are critical residues on promoting their binding with ABBA by forming non-covalent weak interactions. ABBA reduced the cytotoxicity and inflammatory responses mediated by *L. monocytogenes*, and demonstrated protective effect against *L.monocytogenes*-infected *Galleria mellonella*. ABBA did not show anti-*L. monocytogenes* properties and cytotoxicity effect under the test concentrations, promising its potential to be developed as an anti-*L. monocytogenes* infection agent.

## Introduction

*Listeria monocytogenes* is one of the deadliest foodborne pathogens, it can cross the blood-brain barrier and placental barrier after infecting people, causing gastroenteritis, bacteremia, meningitis and many other diseases, the mortality of *L. monocytogenes* infections can reach as high as 30%, which bring expensive medical costs and seriously threaten public health [1-3]. Besides, *L. monocytogenes* also infect chickens, cattle, sheep, pigs and many other animals in the poultry industry to affect their healthy growth, which would reduce the production and quality of poultry industry and cause economic losses [4-6]. *L. monocytogenes* infection is widespread all over the world, the abuse of antibiotic led to *L. monocytogenes* severe resistance, the continuing development of *L. monocytogenes* resistance exerted challenge to veterinary clinical, food safety and public health [7-9]. Therefore, developing alternative strategy and inhibitors to combat this pathogen is urgent.

Listeriolysin O (LLO) is an exocrine toxin of *L. monocytogenes*, it is encoded by the *hly* gene and it belongs to the cholesterol-dependent cytolysin family [10]. LLO can specifically bind to cholesterol on the cell membrane of mammalian to form oligomers that contain 35-40 monomers, the oligomers can pierce the cell membrane, trigger cytotoxicity, and affect the normal physiological functions of cells [11]. LLO also helps *L. monocytogenes* escape from phagocytic vesicles by punching holes on the cell membrane to promote bacteria survival and proliferation [12-14]. LLO have also been reported to trigger inflammatory responses by intervening in host inflammation-related signalling pathways [15, 16]. The *hly* knockout strain was unable to escape phagosomes and could hardly colonise in the spleen and liver of mice [17]. Sortase A (SrtA) is a transpeptidase expressed by *L. monocytogenes*, encoded by the *srtA* gene, which can specifically recognise virulence proteins containing LPXTG motifs, and cleave the peptide bonds between T and G, covalently anchoring virulence proteins to the surface of bacterial cell walls [18]. More than 40 genes encoding LPXTG proteins have been identified in *L. monocytogenes* [19], many of them are internalin family protein, such as InlA protein that helps *L. monocytogenes* adhesion and invasion, the InlH protein that affects host immune response, and the InlL that affects biofilm formation [19, 20]. The *srtA* knockout strain was unable to adhere to and invade host cells, and its pathogenicity was significantly reduced in mouse models [21-23]. Therefore, LLO and SrtA are key targets for the development of novel anti-*L. monocytogenes* agents.

Dryocrassin ABBA (ABBA) is mainly exists in medicinal plant that named *Dryopteris crassirhizoma Nakai* [24]. ABBA has been confirmed to possess anti-inflammatory, anti-cancer, and antibacterial properties [25, 26]. Researches indicated that ABBA has the potential to inhibit the virulence of *Streptococcus pneumoniae* and *Staphylococcus aureus* [24-26]. However, ABBA inhibits *L. monocytogenes* pathogenicity by targeting LLO and SrtA has not been disclosed. Here, we found that ABBA inhibited the hemolytic activity of LLO protein and *L. monocytogenes* culture supernatant by directly binding with LLO and reduced its secretion to the cultural medium. In addition, ABBA inhibited the formation of *L. monocytogenes* biofilm and its adhesion and invasion to host cells by targeting SrtA. Furtherly, ABBA reduced the cytotoxicity and inflammation mediated by *L. monocytogenes* and demonstrated a comprehensive protective effect against *L. monocytogenes*-infected *Galleria mellonella* model. The elucidation of the molecular mechanism of ABBA interacted with LLO and SrtA established the foundation for its application.

## Materials and Methods

### Reagents, Proteins, Cell Lines, Strains, and Growth Conditions

ABBA (purity ≥98%) was purchased from Chengdu Herpurify Co., Ltd. (China). Trypticase Soy Broth (TSB) and agar were purchased from Haibo Bio-Technology Co., Ltd., (China). The *G. mellonella* was purchased from Tianjin Huiyude Biotechnology Co., Ltd., (China). The substrate peptide of SrtA (Dabcyl-QALPETGEE-Edans) was purchased from GL Biochem (China), Ltd. The pET-28a vector that carried the *hly* or *SrtA* gene was constructed in our previous research, the LLO (wild type or its mutants) and SrtA proteins were purified and stored at -80°C based on previously reported methods [27, 28]. *L. monocytogenes* ATCC19115, mouse macrophage J774A.1 and Hela cells were purchased from American Type Culture Collection (ATCC). *L. monocytogenes* was cultured in TSB medium at 37°C with shaking or static.

### LLO and *L. monocytogenes* Culture Supernatant Hemolytic Activity Inhibition

The hemolytic activity assay was carried out as described previously [29]. In brief, purified LLO protein (1 μg) was incubated with different concentrations of ABBA (0, 1.25, 2.5, 5, and 10 μM) at 37°C for 20 min, and then sterile defibrinated sheep blood was added (final concentration 2.5%). After incubation for 10 min, the samples were centrifuged at 12,000 rpm for 2 min, and equal volumes of the supernatant were collected to measure the absorbance at 543 nm (Abs543) to analyze the inhibitory effect of ABBA on LLO activity. For *L. monocytogenes* cultural supernatant hemolytic activity assay, equal volume supernatant was added to sterile phosphate buffered saline (PBS) buffer that contained sterile defibrinated sheep blood (2.5%) and incubated at 37°C for 10 min, then samples were detected Abs543 after centrifuged. Sheep blood cells treated with sterile water or PBS were used as positive control (PC) or negative control (NC).

### Oligomer Formation Inhibition Assay

This assay referred the method reported previously [17]. LLO protein (4 μg) was incubated with with different concentrations of ABBA (0, 10, and 20 μM) at 37°C for 20 min, following sterile defibrillated sheep blood was added with the final concentration of 1%. After incubated on ice for 2 min, sodium dodecyl sulfate-polyacrylamide gel electrophoresis (SDS-PAGE) loading buffer without β-mercaptoethanol was added and all samples were treated at 55°C for 10 min. The samples were separated by using a 6% SDS-PAGE gel, and the proteins were transferred to PVDF membranes. After blocked with 5% skimmed milk, samples were incubated with His-tag antibody for 2 h at room temperature (1:1000, Sangon Biotech, D191001), after washed, the PVDF membranes was incubated with a sheep anti-mouse secondary antibody that conjugated with HRP. Image was collected to analyze the effect of ABBA on the formation of LLO oligomers. The LLO monomer detection was based on coomassie brilliant blue dyeing.

### Computational Biology Assay

This assay was performed by referencing methods described previously [30]. Simply, the crystal structures of LLO (PDB ID 4CDB) and SrtA protein (PDB ID 5HU4) were used as receptors, ABBA (CAS) was used as ligand. A docking box with the spacing of 1 angstrom (Å) was generated, AutoDock Vina was used to perform the docking calculation. After obtaining the complexes, the molecular dynamics simulations assays were performed by using GROMACS 2020.6 to confirm the reliability of the binding. Amber 99SB-ILDH force field and TIP3P water model were applied. Root-mean-square deviation (RMSD) was analyzed to evaluate the stability of the conformations. Binding free energy was analyzed to determine the critical residues involving in the binding. The mutants proteins of LLO that used for calculating the binding free energies were generated by using swiss-PdbViewer 4.10 [31].

### Bacterial Growth Assay

*L. monocytogenes* and various concentrations of ABBA (0, 1.25, 2.5, 5, 10, 20, and 40 μM) were incubated at 37°C for 8 h, the Abs600 values of each sample were determined to evaluate the effect of ABBA on bacterial growth. The minimum inhibitory concentration assay was performed as follows: serious concentrations (0, 30, 40, 50, 60, 70, 80, 90, 100, 110, 120 μM) of ABBA in TSB medium was prepared, then bacteria were added to reach a final concentration of 5 × 10^5^ colony forming units per milliliter (CFUs/ml).

### SrtA Activity Experiment

*L. monocytogenes* SrtA protein with different concentrations of ABBA (0, 10, 20, and 40 μM) was incubated at 37°C for 30 min, then the substrate peptides with fluorophores at both ends were added and samples were incubated at 37°C in the dark. After 60 min, the fluorescence intensity of the samples were measured with the excitation wavelength of 350 nm and emission wavelength of 520 nm. The inhibitory effect of ABBA on the activity of *L. monocytogenes* SrtA was analyzed [32].

### *L. Monocytogenes* Biofilm Formation Inhibition Experiment

*L. monocytogenes* was co-cultured with different concentrations of ABBA (0, 2.5, 5, 10, 20, 40, and 80 μM) at 37°C for 24 h. Then the samples were washed with sterile PBS after the culture medium was discarded, and stained with 0.1% crystal violet for 20 min. Subsequently, samples were washed after the excess crystal violet was discarded and treated with 33% glacial acetic acid. Abs570 values were detected to analyze the effect of ABBA on the formation of *L. monocytogenes* biofilms [33].

### Adhesion and Invasion Experiments

Human colorectal cancer cells (CaCO_2_) were cultured in Dulbecco's Modified Eagle's Medium (DMEM) with 10% fetal bovine serum (FBS) at 37°C with 5% CO_2_. Cells were seeded into 24 well plate with a density of 1 × 10^5^ cell/ml and cultured overnight. The next day, the culture medium was replaced with DMEM containing *L. monocytogenes* and various concentrations of ABBA (0, 10, 40 μM). After 60 min of infection, the culture medium was removed, cells were washed with sterile PBS harvested. After diluting, samples were coated on TSB agar plates and incubated overnight at 37°C. The effect of ABBA on *L. monocytogenes* adhesion was evaluate by analyzing the number of the colonies. For the invasion assay, cells with *L. monocytogenes* and ABBA co-cultured for 1.5 h, then the medium was removed and gentamicin (100 μg/ml) was used to treat cells for 1 h. Following, the medium was removed, cells were lysed after washing, and samples were coated onto TSB medium and cultured overnight [33]. The inhibitory effect of ABBA on *L. monocytogenes* invasion was evaluated by counting the colonies number.

### Detection of Lactate Dehydrogenase Activity

J774A.1 were cultured in DMEM with 10% FBS at 37°C with 5% CO_2_. The cells were seeded into 96 well plate (1.5 × 10^4^ cells/well) and cultured overnight. The next day, *L. monocytogenes* with various concentrations of ABBA (0, 2.5, 5, 10 μM) was used to treat cells. Five hours later, the cultural medium were used to detect the lactate dehydrogenase (LDH) level by referring the protocol of a kit (Beyotime, China). The cells that received 10 μM ABBA treatment were stained with live/dead reagents (Beyotime), images were obtained to observe the survival of the cells [29]. The cytotoxicity of ABBA or it alleviated the cytotoxicity from LLO was determined by detecting the LDH levels.

### Inflammatory Factor Detection Experiment

J774A.1 cells were seeded in a 6-well plate (1 × 10^6^ cells/well) and cultured overnight. The next day, the culture medium was replaced with fresh DMEM containing *L. monocytogenes* and ABBA (0, 5, 10, 20 μM), and samples were treated for 5 hours at the same cultural condition. Then the culture medium was transferred to clean tubes and centrifugated (4°C, 12000 rpm, 5 min)[29]. The levels of tumor necrosis factor α (TNF-α) and interleukin 1β (IL-1β) that released to the medium were detected by using an enzyme-linked immunosorbent assay (ELISA) kit (Sangon Biotech, China).

### *L. monocytogenes* Infect *G. mellonella* Model

*G. mellonella* used in this assay were purchased from Tianjin Huiyude Biotechnology Co., Ltd. Healthy and without melanin samples were used to carry out the assay. *L. monocytogenes* were injected into each *G. mellonella* (2 × 10^5^ CFUs) by using a microinjection pump to construct the infection model, the infected *G. mellonella* were divided into three groups randomly, and nine individual was arranged to each group. 30 min after infection, ABBA (50 mg/kg, 100 mg/kg) was injected into the infected samples, the infected *G. mellonella* that received equal volume solvent treatment was defined as infection group, which was used as positive control (PC), healthy and without melanin samples that got solvent treatment was defined as negative control (NC) group[34]. The survival of the *G. mellonella* were monitored everyday to evaluate the protect effect of ABBA against *L. monocytogenes* infection *in vivo*. Samples from each group was stained with hematoxylin-eosin (H&E) after fixing with 4% formaldehyde, the pathological tissue damage of each sample was observed to determine the alleviation of ABBA against *L. monocytogenes* infection symptoms.

### Data Statistics and Analysis

The research data that obtained from three independent experiments were shown as means with standard deviations (SDs), the analysis of significant differences between data was performed by using GraphPad Prism software (version 9.5.0) which built-in *t* test analytical method, *p* ≤ 0.05 was defined as significant difference.

## Results

### ABBA Inhibits LLO Activity by Binding with and Suppressing Its Oligomer Formation

**Fig. 1:**
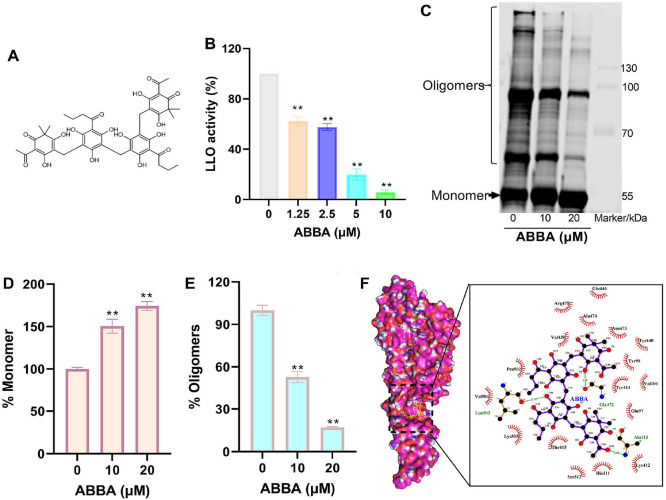
ABBA binds with LLO to inhibit its oligomer formation and reduces its hemolytic activity. (**A**) The molecular structure of ABBA. (**B**) The hemolytic activity of LLO when treated with various concentrations of ABBA. LLO protein was co-incubated with various concentrations of ABBA and aseptic defibrillated sheep red blood cells were added, Abs543 was measured to evaluate the inhibitory effect. Data are present as means with SD, *n* = 3, ** represents *p* ≤ 0.01. (**C**) LLO oligomer formation when it was treated with different concentrations of ABBA and the percentage of monomers (**D**) and oligomers (**E**). LLO protein was treated with different concentrations of ABBA, aseptic de-fibrillated sheep red blood cells were added to induce oligomer formation, image was obtained after the samples were treated with specific antibody, the quantify of the monomer and oligomer was performed by using ImageJ 1.54g. Data are present as means with SD, *n* = 3, ** represents *p* ≤ 0.01. (F) The binding model of LLO and ABBA. AutoDock Vina.

The molecular structure of ABBA is shown in Fig. 1A. The LLO activity in the group that did not receive ABBA was defined as 100%, when various concentrations of ABBA was added to the reaction system, the LLO activity reduced to 62.32%, 57.65%, 19.81% and 5.84% respectively (Fig. 1B). As a member of cholesterol-dependent cytolysin (CDC), forming oligomer is critical for LLO activity, we speculate that ABBA inhibits the formation of LLO oligomers, which was confirmed by the oligomer formation assay. As shown, the LLO monomer was approximately 55 kDa (Fig. S1), the amount of LLO monomer increased along with the increasing ABBA concentrations (100% to 150.52%, 174.29%), but the oligomer formation decreased gradually with the values from 100% to 52.82%, 17.02% (Fig. 1C-1E), suggesting a direct binding between ABBA and LLO was generated, this was confirmed by a docking calculation assay. After docking, nine binding mode was obtained, eight of them were bound at the interface between domains 2 and 4 of LLO, and only one was bound at the interface between domains 1 and 3 (binding mode 6) (Fig. S2), indicating that ABBA is more likely to bind at the junction between domains 2 and 4. The binding mode 1 that with the affinity of -9.97 kcal/mol was shown in Fig. 1F, the binding sites analysis showed that Val504, Pro502, Leu503, Tyr414, Arg475 generated interactions with ABBA (Fig. 1F). These findings suggest that ABBA inhibits oligomer formation by directly binding to LLO proteins, thereby reducing its hemolytic activity.

### ABBA Inhibits the Activity of *L. monocytogenes* Culture Supernatant

**Fig. 2:**
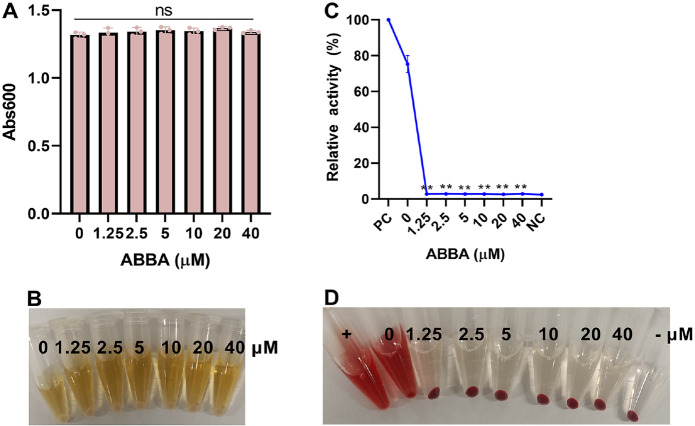
ABBA inhibits the hemolytic activity of *L. monocytogenes* culture supernatants. (**A-B**) The effect of ABBA on *L. monocytogenes* growth and the activity of culture supernatants when *L. monocytogenes* was co-cultured with serious concentrations of ABBA (**C-D**). *L. monocytogenes* was co-cultured with serious concentrations of ABBA for 8 h, the Abs600 was measured to determine whether ABBA affect *L. monocytogenes* growth; the culture supernatants were harvested, equal volume samples were added to PBS buffer that containing sheep red blood cells, Abs543 was detected to determine the hemolytic ability of the samples. Data are present as means with SD, *n* = 3, ns represents no significant.

The bacteria reach the same growth endpoint when *L. monocytogenes* was co-cultured with different concentrations of ABBA for 8 h (Fig. 2A and 2B), indicating that ABBA does not affect the normal growth of *L. monocytogenes* within the test concentration, the CFUs analysis also confirm this result (Fig. S3). The minimum inhibitory concentration of ABBA against *L. monocytogenes* was 100 μM. The hemolytic activity of the *L. monocytogenes* and ABBA cultural supernatant was analyzed, as shown, the amount of heme that released to the medium in the PC group was defined as 100%, the relative activity of *L. monocytogenes* cultural supernatant was 75.27%, while, the hemolytic activity of the samples that received ABBA treatment decreased sharply to 2.78%, which is comparable to the NC group (Fig. 2C-2D).

### Elucidation of the Interactive Mechanism between ABBA and LLO

**Fig. 3:**
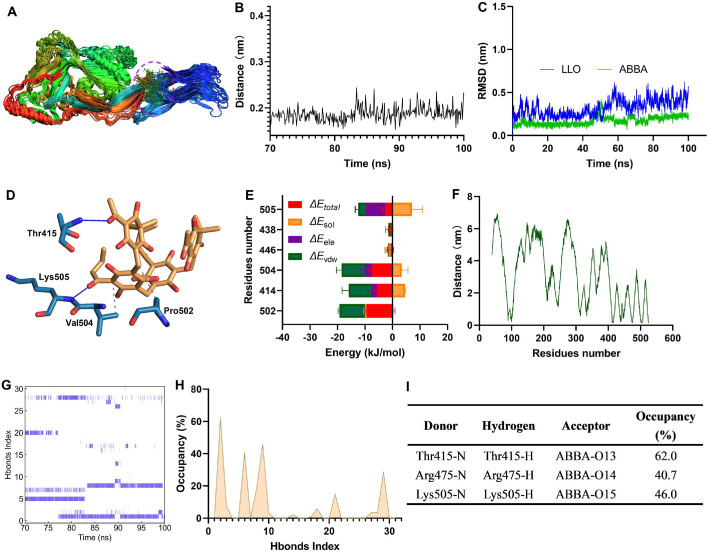
ABBA interacts with Thr415, Lys505, Pro502, and Val504 of LLO. (**A**) The structure superposition of LLO and ABBA at different times during the simulation. (**B**) The distance of ABBA and LLO from the equilibrium trajectory and the RMSD values of ABBA and LLO against the simulation time (**C**). (**D**) The potential binding sites between ABBA and LLO. (**E**) The energy contribution of residues in LLO and the distance between residues of LLO and ABBA (**F**). (**G**) The Hbonds map between ABBA and LLO and their occupancy (**H**) and donor/acceptor datails (**I**). The molecular dynamics simulation assay was performed by using GROMACS 2020.6 version, the indicators analysis were based on the commands built into GROMACS.

To explore the interactive mechanism between LLO and ABBA, we performed molecular dynamics simulation assay. The trajectory files were obtained based on a 100 ns simulation, the structural superposition of ABBA and LLO at different time-points shows that ABBA consistently occupy the initial binding position of LLO during the simulation process (Fig. 3A). The distance between ABBA and LLO fluctuated around 0.2 nm (Fig. 3B), further confirming the stability of the binding. The RMSD of LLO and ABBA fluctuated around 0.32 nm and 0.17 nm, respectively during the simulation process (Fig. 3C), suggesting that they maintained excellent configuration throughout the dynamics process. Thr415, Lys505, Pro502, and Val504 of LLO generated interaction with ABBA (Fig. 3D). These interactions were confirmed by the residue energy decomposition, besides, Tyr414 also contribute more energy (Fig. 3E), the closer distance between these residues and ABBA (Fig. 3F) prove the energy contributions. Hydrogen bonds (Hbonds) analysis reveal more than 30 pairs of Hbonds between ABBA and LLO were generated during 70-100 ns, though many of them have a low existence (Fig. 3G-3H), there are three pairs Hbonds have a higher occupancy (Fig. 3G-3H). The -NH of Thr415, Arg475, and Lys505 interacted with the -O13, -O14, and -O15 in ABBA, with occupancy of 62.0%, 40.7%, and 46.0%, respectively (Fig. 3I). Besides, the binding free energies between ABBA and the LLO mutants and the inhibitory effects of ABBA against the LLO mutants proteins activity decreased significantly (Tables S1 and S2). As shown in Fig. S4, The LLO protein consists of four domains, namely domain 1, 2, 3, and 4. Domain 4 specifically binds to cholesterol of host cells, triggering a series of structural changes in the LLO protein. Subsequently, multiple monomers assemble into a barrel-shaped oligomer, which inserts into the cell membrane, causing leakage of cellular contents and leading to disease. Although it is the fourth domain that interacts with the host receptor, each domain of LLO is crucial for oligomer formation. In this study, ABBA binds at the junction between Domain 2 and Domain 4, where residues Lys505, Pro502, Val504, and Tyr414 form weak interactions that affect the formation of LLO oligomers, thereby inhibiting LLO's hemolytic activity.

### ABBA Inhibits Biofilm Formation, Adhesion and Invasion of *L. monocytogenes* by Affecting SrtA Function

**Fig. 4:**
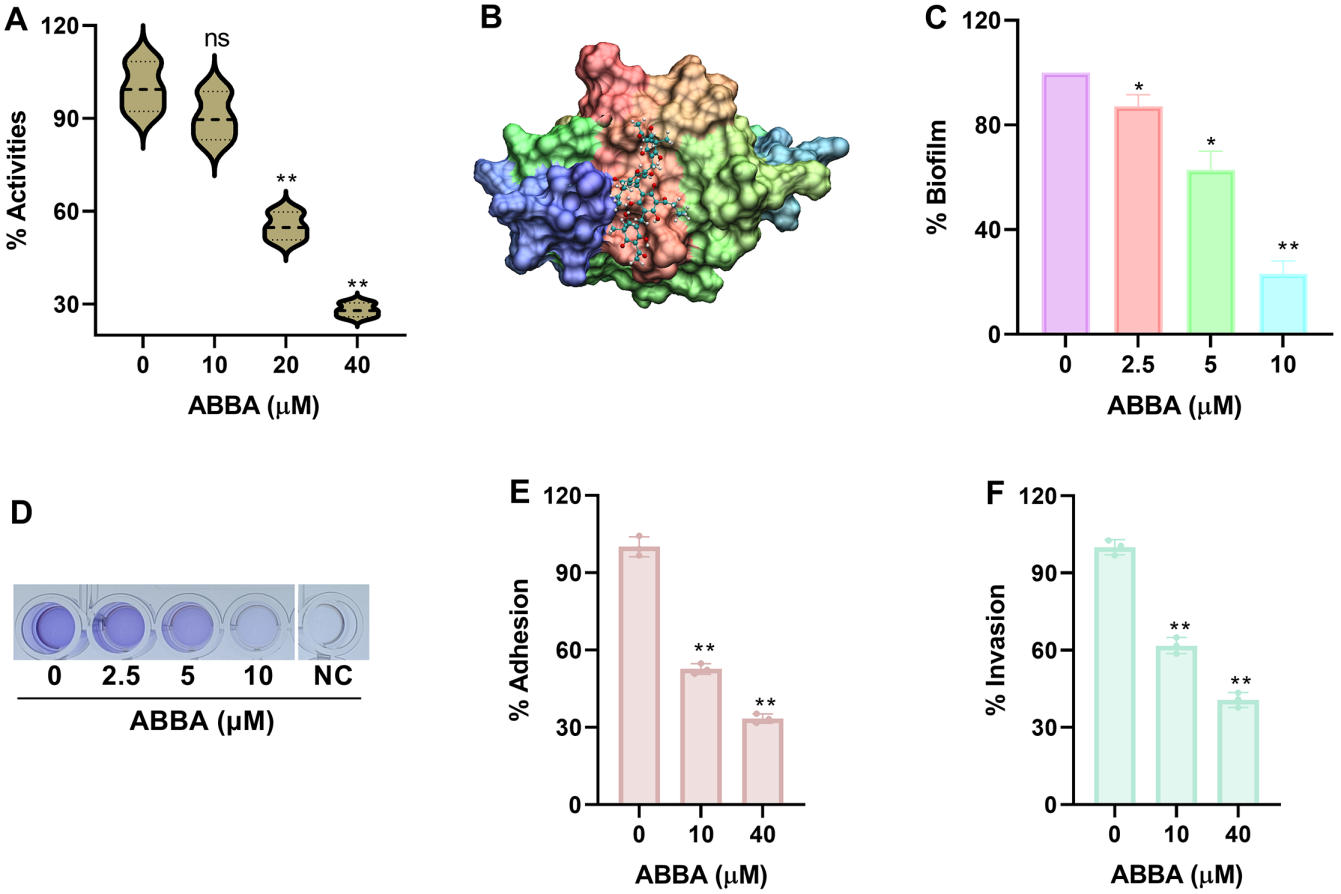
ABBA inhibits *L. monocytogenes* biofilm formation, adhesion and invasion by suppressing SrtA function. (**A**) The transpeptidase activity of SrtA when it was treated with various concentrations of ABBA and the binding model of ABBA and SrtA (**B**). SrtA protein was treated with various concentrations of ABBA, following substrate peptide with fluorescent groups were added, the inhibitory effects of ABBA against SrtA activity was determined by measuring fluorescence intensity. Data are present as means with SD, *n* = 3, ns represents no significant, ** represents *p* ≤ 0.01. (**C**) The biofilm formation when *L. monocytogenes* was treated with serious concentrations of ABBA and the images of crystal violet staining (**D**). *L. monocytogenes* was co-cultured with serious concentrations of ABBA, following samples were treated with crystal violet and acetic acid, the inhibitory effect was determined by detecting Abs570. Data are present as means with SD, *n* = 3, * represents *p* ≤ 0.05, ** represents *p* ≤ 0.01. (**E**) The adhesion and invasion (**F**) of *L. monocytogenes* to host cells when samples were treated with different concentrations ABBA. Caco2 cells were treated with *L. monocytogenes* and different concentrations of ABBA, cells were harvested after washing, samples were cultured on TSB agar medium, clones were used to analyze the effect of ABBA on *L. monocytogenes* adhesion and invasion.Data are present as means with SD, *n* = 3, ** represents *p* ≤ 0.01.

The activity of SrtA from sample that did not receive ABBA treatment was defined as 100%, while, when ABBA was added, the activity of SrtA reduced to 90.50%, 55.12%, and 28.12% respectively (Fig. 4A), suggesting ABBA generated direct interaction with SrtA, this was confirmed by molecular docking assay, the result showed that ABBA bound to the active pocket of SrtA with an affinity of -8.67. Kca/mol (Fig. 4B). Surface proteins that anchored by SrtA involving in *L. monocytogenes* biofilm formation, adhesion and invasion of the bacteria to host cells, therefore, we evaluated these indicators. For biofilm formation, when various concentrations of ABBA was used to treat *L. monocytogenes*, the biofilm formation reduced from 100% to 87.19%, 62.81% and 23.11% (Fig. 4C), the visual image demonstrate the quantitative results (Fig. 4D). For adhesion and invasion assay, we found that when different concentrations of ABBA was used to treat samples, the adhesion of *L. monocytogenes* to Caco2 cells reduced from 100% to 52.71% and 33.45% (Fig. 4E), and the invasion reduced to 61.78% and 40.67% (Fig. 4F). These results indicate that ABBA binds to SrtA and inhibits its biological function, thereby reducing biofilm formation and the adhesion and invasion of bacterial to host cell.

### ABBA Generates Interactions with Asn92, Pro220, Val91 and Lys222

**Fig. 5:**
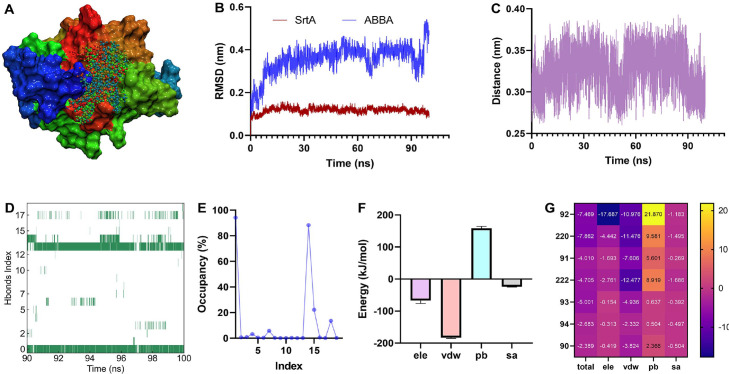
Asn92, Val91, Pro220 and Lys222 of SrtA generate interaction with ABBA. (**A**) The relative position of ABBA against SrtA during the simulation. (**B**) The RMSD fluctuation of ABBA and SrtA and the distance between them (**C**). (**D**) The Hbonds map between ABBA and SrtA and their occupancy (**E**). (**F**) The binding free energy between ABBA and SrtA and the residues energy contribution (**G**).

To elucidate the interactive molecular mechanism between ABBA and SrtA, a 100 ns molecular dynamics simulation experiment was performed, and we found that ABBA was always located in the initial binding pocket of SrtA throughout the simulation process by analyzing the relative positions of ABBA and LLO at different moment (Fig. 5A). During the simulation, the RMSD of ABBA and SrtA fluctuated around 0.36 nm and 0.12 nm, respectively (Fig. 5B), and the distance between ABBA and SrtA fluctuated around 0.33 nm (Fig. 5C), indicating that ABBA and SrtA maintained stable structures and binding modes during the simulation. There are nearly 20 pairs of hydrogen bonds at 90-100 ns (Fig. 5D), but most of them were ephemeral, two pairs were stable existence with the occupancy of 94.20% and 88.30%, respectively (Fig. 5E), the -NH and -O groups of Asn92 interacted with the -O12 and -O16 of ABBA, respectively (Table 1). We found that van der Waals force (vdw) and electrostatic action (ele) are the main interaction forces with the values of -182.68 kJ/mol and -67.28 kJ/mol (Fig. 5F), more exactly, Asn92, Pro220, Val91, Lys222 contributed more ele and vdw to promote the binding (Fig. 5G).

### ABBA Alleviates Cytotoxicity and Inflammation Mediated by *Listeria monocytogenes*

**Fig. 6:**
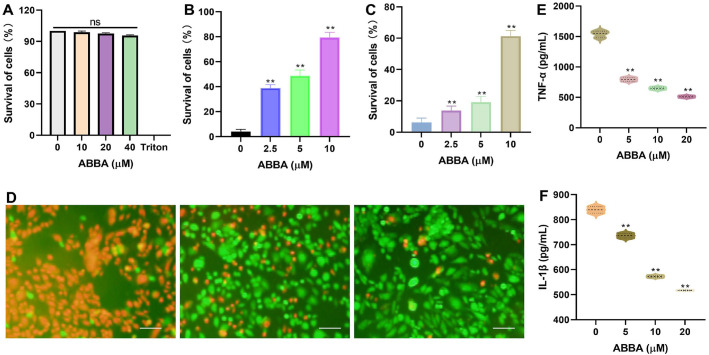
ABBA inhibits the cytotoxicity and inflammation mediated by *L. monocytogenes*. (**A**) The survival of cells when they were treated with ABBA only, LLO and ABBA (**B**) or *L. monocytogenes* with ABBA (**C**). (**D**) The live/dead cells stained when cells were treated with *L. monocytogenes* and ABBA. Cells treated with LLO or *L. monocytogenes* and ABBA was added, the cultural medium were used to detect the levels of LDH that released, the cells were stained with live/dead reagents. Data are present as means with SD, *n* = 3, ns represents no significant, ** represents *p* ≤ 0.01, bar represents 50 μm. (**E**) The levels of TNF-α and IL-1β (**F**) that released to the cultural medium. Cells were treated with *L. monocytogenes* and various concentrations of ABBA, the cultural medium were harvested and the levels of TNF-α and IL-1β were determined by using an ELISA kit. Data are present as means with SD, *n* = 3, ** represents *p* ≤ 0.01.

When J774A.1 cells were treated with various concentrations of ABBA, the survival of the cells was comparable to that of the negative control group (only DMEM treatment group), indicating that ABBA had no cytotoxic effect within the test concentration range (Fig. 6A). For LLO treated cells, the survival was 38.81%, 48.73% and 79.45% when various concentrations of ABBA was applied (Fig. 6B), and the survival was 13.75%, 19.13%, and 61.33% when cells treated with *L. monocytogenes* and ABBA (Fig. 6C). For live/dead cells stain, when different concentrations of ABBA were applied, the red fluorescence significantly decreased, whereas the green fluorescence significantly increased, indicating that dead cells decreased and live cells increased (Fig. 6D). The above results demonstrate that ABBA can significantly alleviate the cytotoxic effects mediated by LLO or *L. monocytogenes* from both qualitative and quantitative perspectives. For ELISA assay, when J774A.1 cells were treated with *L. monocytogenes*, a large amount of TNF-α (1540.07 pg/ml) and IL-1β (839.26 pg/ml) were detected in the culture medium, and the levels of TNF-α decreased to 795.66 pg/ml, 647.95 pg/ml, and 511.32 pg/ml (Fig. 6E), respectively. The level of IL-1β decreased to 736.16 pg/ml, 572.03 pg/ml, and 516.86 pg/ml (Fig. 6F), indicating that *L. monocytogenes* can trigger an inflammatory response in J774A.1 cells, while ABBA can significantly alleviate *L. monocytogenes*-mediated cellular inflammatory responses.

### ABBA Protects *G. mellonella* from *L. monocytogenes* Infection

**Fig. 7:**
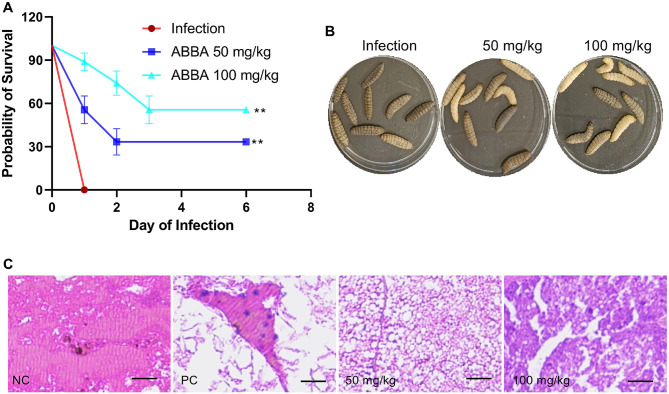
ABBA protects *G. mellonella* from *L. monocytogenes* infection. (**A**) The survival of *G. mellonella* and their infective symptoms (**B**) from different treatment groups. *G. mellonella* were treated with *L. monocytogenes* and different concentrations of ABBA, each group contain nine *G. mellonella*, the survival and the symptoms of the samples were observed, ** represents *p* ≤ 0.01. (**C**) The pathological damage of the *G. mellonella* tissue from different treatment groups. Samples were fixed with formaldehyde and stained with H&E, images were obtained by using an optical microscope. Bar represents 200 μm.

All the *G. mellonella* in the infected group died at 24 h, the 50 mg/kg and 100 mg/kg ABBA treatment groups also found died *G. mellonella* at 24 h, but the survival of these groups were 33.33% and 55.56%, respectively (Fig. 7A). For the infectious symptoms, individual in the infection group died completely, they were swelling and the whole body was filled with melanin, but these characters were alleviated in the samples from the ABBA treatment group (Fig. 7B). Sample from the NC group exhibited complete, undamaged tissue structure, and there was no inflammatory infiltration and melanin; samples from the PC group showed complete tissue destruction and infiltration of inflammatory factors, while, these characters were alleviated in the ABBA treatment group samples (Fig. 7C). These results indicate that *L. monocytogenes* infection could cause serious tissue damage, trigger inflammation, and result in death, whereas ABBA treatment could significantly alleviate pathological tissue damage, reduce the inflammatory response, and improve the survival of the infected *G. mellonella*.

## Discussion

LLO serves as the “Swiss army knife” of *L. monocytogenes* [35, 36], considering its crucial role in *L. monocytogenes* infection, research on its inhibitors is compelling. Some bioactive properties from medicinal plants have been identified as inhibitors of LLO, such as amentoflavone, atractylodin, fisetin, and so on [37-39]. These inhibitors suppress *L. monocytogenes*-mediated inflammatory responses and their pathogenicity by intervening in LLO function [40]. But reports on plant activity ingredients targeting both LLO and SrtA are rare, phloretin and isoflavone glucoside genistin showed inhibiting effect to these toxins [28, 41]. This study found that ABBA simultaneously targets LLO and SrtA to suppress *L. monocytogenes* virulence. By targeting LLO and SrtA, ABBA reduced the haemolytic activity of *L. monocytogenes* culture supernatant and biofilm formation, and it inhibited the adhesion and invasion of *L. monocytogenes* to the host cells. We speculate that the inhibition of SrtA function results in a reduction of surface protein that anchored to the bacterial cell wall, such as inlA, which lead to the reduction of bacterial adhesion and invasion to cells, which may be explored in our future work.

The crystal structures of LLO and SrtA have been elucidated [42, 43]. The LLO crystal structure is divided into four domains; the residues in domain four interact with the host cell receptor and form oligomer to pierce cells. However, previous studies have demonstrated that the other three domains are crucial for the formation of LLO oligomers [42, 44]. The binding sites of previously reported inhibitors with LLO differ slightly; but they can inhibit the formation of LLO oligomers and affect its haemolytic activity [17, 29]. This confirms the importance of different domains in the formation of LLO oligomers and their haemolytic activity. In this study, based on molecular dynamics simulation analysis, Thr415, Lys505, Pro502, and Val504 in LLO protein generated interactions with ABBA and promote their binding. For SrtA, His127, Arg197 and Cys188 have been identified as its active sites that interact with the substrates, as SrtA lost almost all transpeptide activity after these residues were mutated to Ala [45, 46]. In this study, we found that ABBA bound to the back of the active pocket of *L. monocytogenes* SrtA, Asn92, Pro220, Val91 and Lys222 are important residues on promoting their binding. Unlike previous reports, ABBA did not directly bind to *L. monocytogenes* SrtA activity center in this study, and we speculate that ABBA may affect SrtA function through non-competitive inhibition, which worth further exploring in the future.

ABBA did not exhibited anti-*L. monocytogenes* and cytotoxicity effects at the effective inhibiting concentrations. Moreover, ABBA treatment significantly reduced *L. monocytogenes*-mediated cytotoxicity and inflammatory responses, and it protected *G. mellonella* from *L. monocytogenes* infection, which results from the inhibitory effects of ABBA against LLO and SrtA simultaneously, although there may be other underlying mechanisms need to be explore in future. The bioavailability of ABBA can reach as high as 50.1%, and its metabolic kinetics parameters have been well-defined [47]. These results provide a significant basis for future applications of ABBA.

## Conclusion

ABBA, a natural active ingredients, inhibited the hemolytic activity of LLO and *L. monocytogenes* culture supernatants by suppressing oligomer formation. In addition, ABBA directly bound with SrtA to inhibit its transpeptidase activity, which resulted in a reduction of bacterial cell wall surface proteins that anchored by SrtA, thus the formation of bacterial biofilm was defective, and the adhesion and invasion of bacteria to host cell were alleviated. ABBA and LLO or SrtA maintained stable and reliable binding model, weak interaction such as Hbonds, vdw and ele were generated during the interaction. ABBA did not exhibit anti-*L. monocytogenes* or cytotoxicity effects at the effective inhibition concentrations, but it alleviated the cytotoxicity and inflammation mediated by LLO or bacteria, and protected *G. mellonella* from *L. monocytogenes* infection. These research results provide evident for the application of ABBA on combating *L. monocytogenes* infection.

## Supplemental Materials

Supplementary data for this paper are available on-line only at http://jmb.or.kr.



## Figures and Tables

**Table 1:** The Hbonds details between ABBA and SrtA.

Donor	Hydrogen	Acceptor	Occupancy (%)
Asn92-N	Asn92-H	ABBA-O16	94.20
ABBA-O12	ABBA-H97	Asn92-O	88.30
