# Supplementary material for: Dryocrassin ABBA Inhibits the Function of LLO and SortaseA to Alleviate the Virulence of Listeria monocytogenes
Source: J Microbiol Biotechnol. 2026 Jan 26;36:e2510021. doi: 10.4014/jmb.2510.10021 (PMC12868946; doi:10.4014/jmb.2510.10021)
Supplement: Supplementary file 1 [file jmb-36-e2510021-supple.pdf]

Supplementary files for

Dryocrassin ABBA inhibits the function of LLO and SortaseA to alleviate the virulence of *Listeria monocytogenes*

Jiahui Lu, Junlu Liu, Hanbing Zhou, Yifan Duan, Zehua Wang, Guizhen Wang*

College of Biological and Food Engineering, Jilin Engineering Normal University, Changchun, China 130052

*Correspondence: Guizhen Wang: (wanggz@jlenu.edu.cn), Jilin Engineering Normal University, phone: 18604303651, fax numbers: 0431-86908203

Running title: ABBA anti-*L. monocytogenes* infection

Key words: Anti-virulence, Anti-infection, Sortase A, LLO, Dryocrassin ABBA

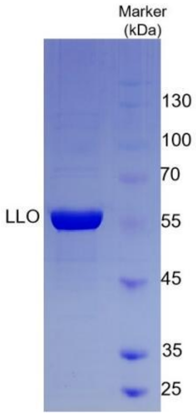

Figure S1 The image of LLO monomer protein. The purified LLO protein was separated by 10% SDS-PAGE gel, then the protein was stained with Coomassie brilliant blue and the image was obtained.

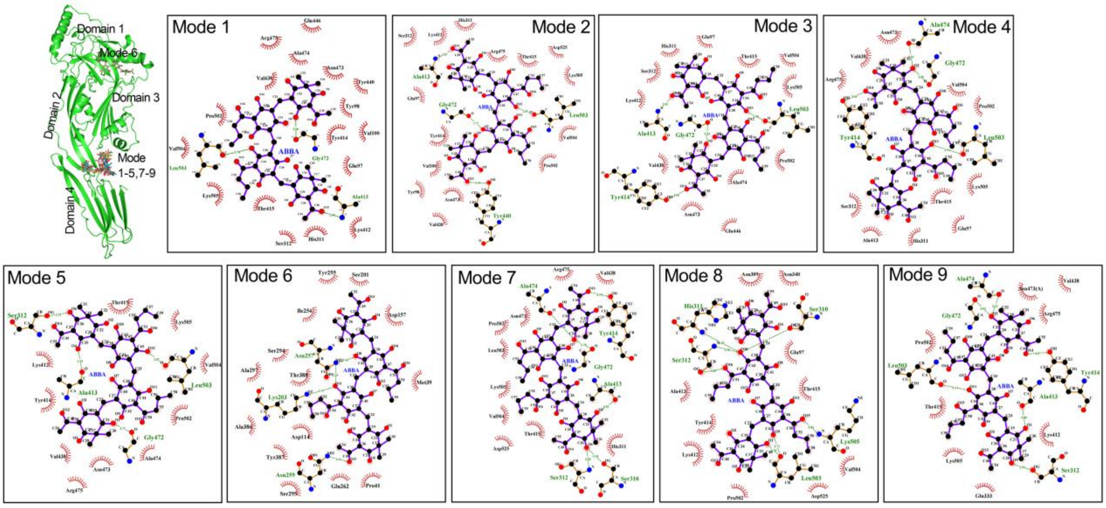

Figure S2 The binding mode and the interface annotations. There are nine potential binding modes between ABBA and LLO, eight of them were bound at the interface between domains 2 and 4, and only one was bound at the interface between domains 1 and 3 (binding mode 6). The interface annotations of these nine binding

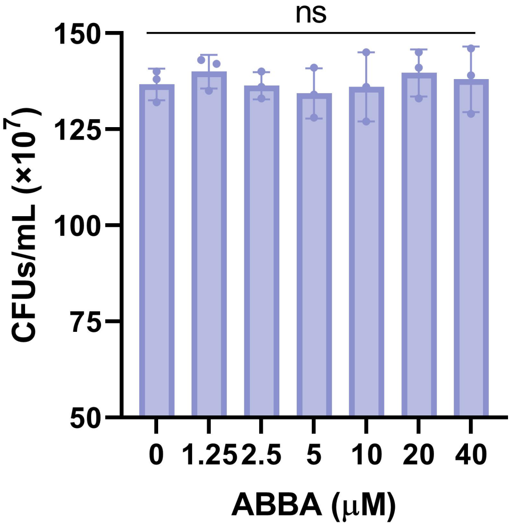

Figure S3 ABBA does not affect the growth of *L. monocytogenes*. *L. monocytogenes* was co-cultured with various concentrations of ABBA until reached to plateau stage, samples from each group were harvested and plated onto TSB agar after dilution. After cultured overnight, the clones were obtained to analyze the effect of ABBA on the growth of *L. monocytogenes.* Data were shown as means with SDs, n=3, ns represents no significant.

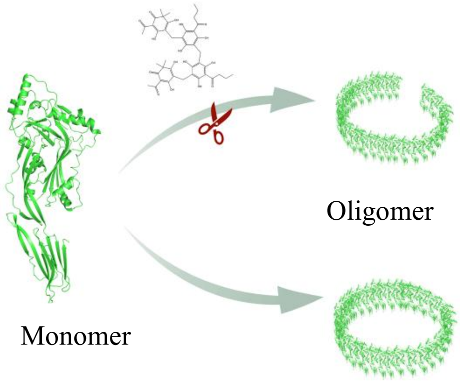

Figure S4 The structural model of the LLO oligomer. The LLO protein consists of four domains, namely domain 1, 2, 3, and 4. Domain 4 specifically binds to cholesterol of host cells, triggering a series of structural changes in the LLO protein. Subsequently, multiple monomers (approximately 30-40) assemble into a barrel-shaped oligomer, which inserts into the cell membrane, causing leakage of cellular contents and leading to disease. Although it is the fourth domain that interacts with the host receptor, each domain of LLO is crucial for oligomer formation. ABBA binds at the junction between domain 2 and domain 4, where residues Lys505,

Arg475, Thr415, Pro502, Val504, and Tyr414 form weak interactions that affect the formation of LLO oligomers, thereby inhibiting LLO's hemolytic activity. mode were shown.

Table S1 The binding free energies between ABBA and LLO or its mutants

**Index**	**Protein**	**Energy（kJ/mol）**	**Significance**
	ABBA-LLO$_{WT}$	-82.41 ± 2.31	
	ABBA-LLO$_{V504A}$	-44.38 ± 2.21	**
ΔE_{total}	ABBA-LLO$_{K505A}$	-59.54 ± 0.97	**
	ABBA-LLO$_{P502A}$	-47.80 ± 2.03	**
	ABBA-LLO$_{Y414A}$	-49.14 ± 3.97	**
	ABBA-LLO$_{WT}$	-43.12 ± 6.70	
	ABBA-LLO$_{V504A}$	-16.80 ± 1.13	*
ΔE_{ele}	ABBA-LLO$_{K505A}$	-21.02 ± 5.21	*
	ABBA-LLO$_{P502A}$	-27.96 ± 2.37	ns
	ABBA-LLO$_{Y414A}$	-31.23 ± 2.39	ns
	ABBA-LLO$_{WT}$	-128.05 ± 3.99	
	ABBA-LLO$_{V504A}$	-85.94 ± 6.26	**
ΔE_{vdw}	ABBA-LLO$_{K505A}$	-109.54 ± 2.14	**
	ABBA-LLO$_{P502A}$	-95.97 ± 3.44	**
	ABBA-LLO$_{Y414A}$	-98.02 ± 3.89	**

Note:* represents $p < 0.05$, ** represents $p < 0.01$, ns represents no significance

Table S2 The inhibitory effects of ABBA against LLO or its mutants

**Inhibitor**	**Protein**	**Inhibition（%）**	**Significance**
	LLO$_{WT}$	94.16 ± 1.45	
	LLO$_{V504A}$	52.47 ± 3.78	**
ABBA 10 μM	LLO$_{K505A}$	73.62 ± 2.13	**
	LLO$_{P502A}$	60.25 ± 4.09	**
	LLO$_{Y414A}$	69.58 ± 1.86	**

Note:** represents $p < 0.01$
